# Deciphering the Role of Insulin-Like Growth Factor-I Receptor in Trastuzumab Resistance

**DOI:** 10.1155/2012/648965

**Published:** 2012-07-09

**Authors:** Rita Nahta

**Affiliations:** Departments of Pharmacology, Hematology and Medical Oncology, School of Medicine; Winship Cancer Institute; Molecular and Systems Pharmacology Program, Graduate Division of Biological and Biomedical Sciences, Emory University, Suite 5001, 1510 Clifton Road, Atlanta, GA 30322, USA

## Abstract

Resistance to the HER2-targeted antibody trastuzumab is a major clinical concern in the treatment of HER2-overexpressing metastatic breast cancer. Increased expression or signaling of the insulin-like growth factor-I receptor (IGF-IR) has been reported in a subset of cell lines and clinical samples derived from trastuzumab-resistant breast cancers. Genetic and pharmacologic inhibition of IGF-IR signaling has been shown to improve response to trastuzumab in trastuzumab-naïve and trastuzumab-resistant models. In this paper, we will discuss the role of IGF-IR signaling in trastuzumab resistance. Further, we will discuss cotargeting IGF-IR and HER2 as a potential therapeutic strategy for HER2-over-expressing breast cancers that have progressed on trastuzumab treatment.

## 1. Introduction

Trastuzumab (Herceptin; Genentech, San Francisco, CA) is a humanized monoclonal antibody against an epitope in the extracellular domain of the HER2 receptor tyrosine kinase protein [1]. HER2 is overexpressed, generally due to amplification of the *her2* gene, in approximately 20–30% of human metastatic breast cancers (MBC), and is associated with reduced disease-free survival [2]. Trastuzumab effectively elicits pathologic complete responses in a large percentage of patients with HER2-positive MBC [3, 4], particularly when combined with chemotherapy [5–7]. However, some patients do not respond to trastuzumab [3–7], displaying so-called primary, *de novo*, or intrinsic resistance. In addition, median duration of response to trastuzumab-based therapy was reported to be less than one year in initial trials [3–7], indicating that acquired resistance is a common clinical concern. A clearer understanding of the mechanisms that contribute to trastuzumab resistance is needed in order to develop new therapeutic strategies, and, ultimately, to improve survival outcomes for patients with HER2-over-expressing breast cancer.

## 2. Preclinical Studies Examining IGF-IR and Trastuzumab Resistance

Multiple molecular mechanisms driving trastuzumab resistance have been proposed. These mechanisms and potential treatment strategies to overcome resistance are discussed in detail in several recent and outstanding reviews [8–12]. In this paper, we will focus on one of the proposed mechanisms of trastuzumab resistance: increased signaling from the insulin-like growth factor-I receptor (IGF-IR).

Lu et al. [13] first provided data to support a possible role for IGF-IR in trastuzumab resistance. The authors [13] showed that trastuzumab effectively activated a G1 arrest response in the HER2-over-expressing breast cancer cell line SKBR3, which expresses a relatively low level of IGF-IR. However, stable over-expression of IGF-IR prevented the trastuzumab-mediated growth arrest response in SKBR3 cells. A separate report [14] confirmed these findings by showing that 10 *μ*g/mL trastuzumab reduced proliferation of SKBR3 cells by 25%, while proliferation of SKBR3/IGF-IR stable transfectants was unaffected by as much as 60 *μ*g/mL of trastuzumab. Under anchorage-independent conditions, trastuzumab inhibited colony growth of SKBR3 empty vector control cells by 62% versus only 12% inhibition in SKBR3 IGF-IR stable transfectants [13]. In addition, MDA231 breast cancer cells transfected with HER2 showed downregulation of IGF-IR and increased sensitivity to trastuzumab [15]. When these MDA231/HER2 cells were chronically exposed to trastuzumab, resistant clones developed. These MDA231/HER2 trastuzumab-resistant cells showed 3-fold higher expression of IGF-IR versus the MDA231/HER2 cells [15]. In addition, imaging studies showed that radiolabeled IGF-I could identify HER2-positive breast cancer cells that were resistant to trastuzumab and overexpressed IGF-IR [15], suggesting that IGF-IR expression may be used as a proxy to identify resistant cancers.

These preclinical studies led to the concept that IGF-IR over-expression may be associated with reduced response to trastuzumab. In support of this concept, Jerome et al. [16] examined IGF-IR levels in a cell line that had acquired resistance to trastuzumab. IGF-IR expression was up-regulated by approximately 3-fold in BT474 trastuzumab-resistant cells relative to the trastuzumab-sensitive parental BT474 line. Thus, chronic exposure to trastuzumab may result in upregulation of IGF-IR in association with resistance. In addition to acquired resistance, IGF-IR expression may be linked to intrinsic resistance to trastuzumab. Phospho-proteomic analysis was performed on the JIMT-1 HER2-positive cell line, which shows primary resistance to trastuzumab [17]. This analysis established that IGF-IR was constitutively activated in this cell line. Activated IGF-IR was localized primarily at focal adhesion structures within the cells.

**Figure 1:**
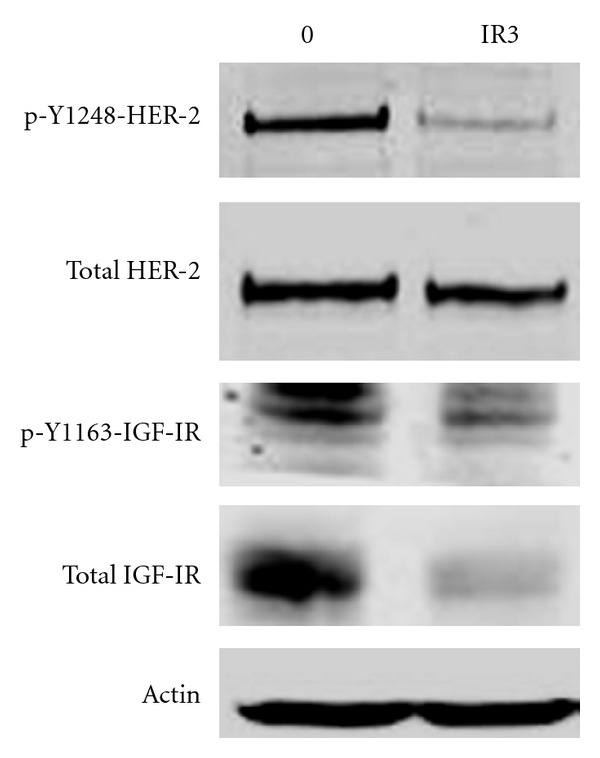
IGF-IR blockade reduces phosphorylation of HER2 in trastuzumab-resistant cells. SKBR3 cells with acquired resistance to trastuzumab were untreated or treated with 0.25 *μ*g/mL alpha IR3 IGF-IR monoclonal antibody overnight. Total protein lysates were western blotted for phosphorylated and total HER2 and IGF-IR. Actin served as a loading control.

We have also analyzed our models of acquired trastuzumab resistance to determine if IGF-IR was overexpressed. Resistant cells were developed by chronically culturing SKBR3 cells in trastuzumab and isolating resistant cells [18, 19]. In comparison to the parental SKBR3 cells, acquired trastuzumab-resistant SKBR3 cells did not show increased IGF-IR expression [20]. However, we identified a novel complex formation between IGF-IR and HER2 in resistant cells, which was not detected in trastuzumab-sensitive parental cells. Huang et al. [21] confirmed this IGF-IR/HER2 interaction and showed that HER3 is also found in this receptor complex. Thus, unique protein-protein interactions occur between HER2 and other receptors, including IGF-IR and HER3, in cells with trastuzumab resistance. Our work showed that this unique interaction facilitates crosstalk from IGF-IR to HER2, such that IGF-I stimulation not only induces phosphorylation of IGF-IR, but also activates phosphorylation of HER2 [20]. IGF-I stimulation in the presence of an IGF-IR kinase inhibitor blocked this crosstalk [20], confirming that the crosstalk occurs from IGF-IR to HER2 and not through another mechanism. Further support for the concept of IGF-IR/HER2 crosstalk in trastuzumab-resistant cells was obtained by blocking IGF-IR with monoclonal antibody alpha IR3 (Figure 1). When resistant cells were treated with alpha IR3 for 1 hour, total IGF-IR levels were unaffected [20]. However, IGF-IR expression was down-regulated when resistant cells were cultured with alpha IR3 overnight (Figure 1). In association with reduced IGF-IR, phosphorylation of HER2 was also suppressed, although total HER2 levels were unaltered. Thus, stimulation or inhibition of IGF-IR resulted in an induction or suppression of HER2 phosphorylation, respectively, in cells with acquired trastuzumab resistance. These data are in direct support of IGF-IR crosstalk to HER2 in the context of trastuzumab resistance.

## 3. Clinical Studies Examining IGF-IR and Trastuzumab Resistance

Despite intriguing preclinical results suggesting a role for IGF-IR in trastuzumab resistance, the translational significance of IGF-IR over-expression or crosstalk to HER2 remains unclear. In two clinical correlative studies, no association was found between IGF-IR expression alone and trastuzumab response [22, 23]. However, increased IGF-IR expression plus increased downstream mTOR signaling correlated significantly with reduced response to trastuzumab [23]. Further, in a recent multivariate analysis [24], high IGF-IR expression was associated with poor prognosis specifically in the HER2-positive subtype of breast cancers. Harris et al. [25] found a correlation between high IGF-IR expression and poor response to preoperative trastuzumab plus chemotherapy. Increased IGF-IR membrane staining, measured by IHC, was associated with lower response to preoperative trastuzumab plus vinorelbine, with a 50% median response rate in the high IGF-IR group versus 97% in the low IGF-IR group [25]. Gallardo et al. [26] performed IHC for several proteins on tumor tissues obtained from patients with early stage or metastatic HER2-positive breast cancer treated with trastuzumab. Amongst 67 patients with early stage disease, IGF-IR over-expression or phosphorylation (inactivation) of the pro-apoptotic protein Bad correlated significantly with shorter progression-free survival (PFS). Amongst 75 patients with metastatic disease, decreased PFS correlated with increased PI3K signaling, and overall survival correlated with vascular invasion and EGFR over-expression. For all samples, staining of IGF-IR was high in 25% of tumors (34/138), which was significantly associated (*P*-value less than or equal to 0.005) with high grade, high mitotic index, and vascular invasion [26]. Thus, a role for IGF-IR in trastuzumab resistance appears to be supported by several clinical correlative studies and should be further studied in this context.

## 4. Potential Mechanisms of IGF-IR-Mediated Trastuzumab Resistance

**Figure 2:**
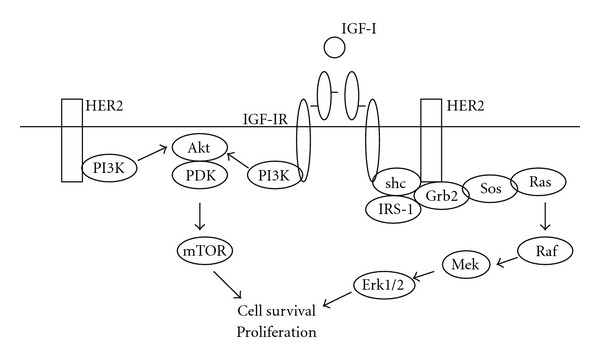
IGF-IR signaling pathway. Increased expression of IGF-IR, or IGF-IR interaction and crosstalk with HER2 have been reported in models of Herceptin resistance. PI3K signaling has been implicated as a potential mechanism of IGF-IR-mediated Herceptin resistance. Inhibition of IGF-IR has been shown to increase sensitivity to Herceptin in preclinical studies.

IGF-IR consists of two alpha and two beta subunits synthesized from a single mRNA precursor. The subunits are cross-linked by cysteine bonds. The alpha chains are extracellular and form the ligand-binding domain, while the transmembrane beta regions possess intrinsic tyrosine kinase activity. IGF-IR tyrosine kinase is activated upon binding IGF-I, inducing autophosphorylation of the receptor (Figure 2). The insulin receptor substrate (IRS) and Shc adaptor proteins, which are bound to the IGF-IR beta subunits, will then become phosphorylated. These proteins couple to and activate downstream signaling through Ras-Raf-MEK-Erk, resulting in increased proliferation. IRS proteins also couple to PI3K, activation of which converts PIP2 to PIP3, and recruits Akt to the plasma membrane where it is activated by PDK. Activated Akt promotes proliferation, cell survival, and migration [27], in part due to activation of mTOR signaling, which increases protein translation.

The mechanisms by which IGF-IR promotes trastuzumab resistance remain largely unknown, although some studies have implicated a role for PI3K signaling. Increased PI3K signaling has been shown to reduce response to trastuzumab in multiple preclinical models [28–31], and was shown to be correlated with clinical trastuzumab resistance [28, 30, 32]. Mechanisms resulting in increased PI3K signaling have primarily focused on downregulation of the phosphatase PTEN and hyper-activating mutations in *PIK3CA*; however, increased growth factor signaling, such as through IGF-IR, will also result in increased PI3K signaling. IGF-I stimulation of cells with acquired trastuzumab resistance induced phosphorylation of IRS-1, HER2, Akt, and Erk1/2 with reduced expression of p27kip1 [20]. Tyrosine kinase inhibition or antibody blockade of IGF-IR blocked phosphorylation of HER2, Akt, and Erk1/2. In MCF7/HER2 and SKBR3/IGF-IR stably transfected cells, IGF-I stimulation blocked trastuzumab-mediated inhibition of Akt and Erk1/2 phosphorylation [16]. A pharmacological inhibitor of phosphoinositide-dependent kinase-1 (PDK-1), OSU-03012 increased trastuzumab-mediated growth inhibition in SKBR3/IGF-IR cells through downregulation of PDK-1/Akt signaling [14]. In addition to PI3K signaling, IGF-IR is likely to promote resistance via activation of mTOR. Inhibition of mTOR has been effective in restoring sensitivity to trastuzumab in a variety of settings [33–35]. Combination trastuzumab plus rapamycin reduced colony formation and invasion in multiple HER2-positive breast cancer cell lines including those with high endogenous IGF-IR expression [36]. Interestingly, a recent clinical correlative study showed that mTOR was overexpressed by IHC in 23% of tumors from patients treated with trastuzumab, in association with IGF-IR over-expression in 47% of the tumors, p110 subunit of PIK3CA in 64% of tumors, and phosphorylation (inactivation) of proapoptotic Bad in 65% of tumors [26]. Thus, in almost half of the tumors that overexpressed mTOR, there was also a potential role for upregulation of IGF-IR-Akt-Bad phosphorylation.

Downstream events resulting in altered expression and function of cell cycle regulators appear to mediate the ultimate increase in proliferation and cell survival propagated by increased IGF-IR signaling. Stable over-expression of IGF-IR resulted in reduced expression of the cyclin-dependent kinase inhibitors p27kip1 and p21cip1 and increased expression of cyclin E [13]. Similarly, trastuzumab-resistant cells have been reported to show reduced p27kip1 [19] and increased cyclin E expression [37]. We showed that cells with acquired resistance to trastuzumab expressed reduced levels of p27kip1 protein [19] but not transcript [38]. Thus, downregulation of p27kip1 appears to occur through posttranslational mechanisms. P27kip1 is known to be largely regulated by phosphorylation and ubiquitination events. IGF-I stimulation in SKBR3/IGF-IR stable transfectants resulted in increased expression of a p27kip1 ubiquitin ligase, Skp2 [39]. We showed that the proteasome inhibitor MG132 induces p27kip1 expression in cells with acquired trastuzumab resistance back to parental levels [19]. Further, resistant cells showed increased sensitivity to MG132 versus parental cells. IGF-I was shown to increase ubiquitination of p27kip1 protein in SKBR3/IGF-IR cells [39]. These results suggest that a potential mechanism by which IGF-IR promotes trastuzumab resistance is via increased ubiquitination and downregulation of p27kip1 protein, resulting in reduced growth arrest and increased proliferation. In fact, reduced p27kip1 expression in resistant cells was associated with increased cdk2 activity and an increased fraction of cells in S phase (proliferation) [19]. Transfection of p27kip1 increased sensitivity to trastuzumab [19], suggesting that downregulation of this downstream protein is an important mechanism of resistance.

Scaltriti et al. [37] showed that amongst 34 patients with HER2-positive breast cancer, *cyclin E* amplification and over-expression was associated with poor clinical benefit to trastuzumab (33.3% compared with 87.5% in those without amplification) and lower progression-free survival (6 months versus 14 months). Over-expression of cyclin E was associated with higher cdk2 activity, and cdk2 inhibition reduced growth of trastuzumab-resistant cell xenografts [37]. Thus, mechanisms downstream of increased IGF-IR signaling, including reduced p27kip1 and increased cyclin E expression, both of which result in increased cdk2 activity, have been reported in trastuzumab-resistant cells.

## 5. Role of Insulin-Like Growth Factor-I-Binding Proteins (IGFBPS) in Trastuzumab Resistance

The IGF-I signaling family includes at least 6 human IGF-binding proteins (IGFBPs). Some IGFBPs bind and sequester IGF-I such that the ligand is unable to bind and activate its receptor. Studies suggest that increased circulating IGFBP levels (particularly IGFBP3) may be used as a marker of increased IGF-IR signaling and trastuzumab resistance; others show that increased expression of IGFBP3 abrogates IGF-IR signaling and increases sensitivity to trastuzumab.

Increased expression of recombinant human IGFBP3 improved response to trastuzumab in multiple models of resistance [13, 16]. In one study [13], MCF7/HER2 stable transfectants, which express high levels of IGF-IR, were not inhibited by trastuzumab in soft agar conditions. IGFBP3 alone inhibited growth by 29%, whereas the combination of trastuzumab plus IGFBP3 inhibited growth by 82%. Similarly, SKBR3/IGF-IR stable transfectants, which were resistant to trastuzumab, showed growth inhibition when cotreated with trastuzumab plus IGFBP3 [13]. Synergy between IGFBP3 and trastuzumab was confirmed by statistical analysis of drug combination dose-effects in SKBR3/IGF-IR, MCF7/HER2, and BT474 acquired resistant cells, but not in parental cells [16]. IGFBP3 suppressed IGF-I signaling in these cell line and xenograft models of resistance [13, 16, 40]. Tumor growth of MCF7/HER2 xenografts was not inhibited by single-agent trastuzumab, whereas single-agent IGFBP3 showed a trend toward growth inhibition [16]. Combined IGFBP3 and trastuzumab treatment resulted in a statistically significant reduction in MCF7/HER2 xenograft tumor volume. IHC analysis of tumor samples showed that Akt and Erk1/2 phosphorylation was maintained at control levels in the trastuzumab-treated group, whereas IGFBP3 (alone or in combination with trastuzumab) reduced Akt and MAPK signaling.

Dokmanovic et al. [41] further suggested that elevated levels of IGFBP3 may reduce IGF-IR/HER2 crosstalk. They showed that trastuzumab induced expression and secretion of IGFBP3 and IGFBP2 in SKBR3 cells in association with growth inhibition. Increased IGFBP3 levels resulted in reduced IGF-I-mediated phosphorylation of IGF-IR, HER2, Akt, and Erk1/2. Further, cells with acquired or intrinsic resistance showed reduced levels of IGFBP3. In contrast, IGFBP2 stimulated phosphorylation of HER2, which was reduced by trastuzumab treatment. Transient transfection of an IGFBP3 expression plasmid into SKBR3 parental or acquired trastuzumab-resistant cells resulted in reduced cell viability [41].

These studies indicate that reduced expression of an endogenous negative regulator of IGF-I activity, IGFBP3, may serve as an indicator of IGF-I signaling and trastuzumab resistance. Strategies that deliver IGFBP3 as a therapy may benefit breast cancers that are resistant to trastuzumab and show elevated IGF-IR signaling.

## 6. IGF-IR Inhibition as a Strategy to Improve Response to Trastuzumab

 Due to preclinical and clinical data suggesting that IGF-IR signaling reduces response to trastuzumab, therapeutic strategies that co-target IGF-IR and HER2 have been studied in models of HER2-over-expressing breast cancer. We showed that the IGF-IR monoclonal antibody (mAb) alpha IR3 restored sensitivity to trastuzumab in models of acquired trastuzumab resistance, in association with disruption of IGF-IR/HER2 dimerization [20]. IGF-IR tyrosine kinase inhibitor (TKI) AG538 also produced dose-dependent reductions in survival of resistant cells [20]. In contrast, trastuzumab-sensitive BT474 cells showed little response to single-agent alpha IR3 or IGF-IR TKI AG1024 [42]. However, combining these IGF-IR inhibitors with trastuzumab or HER2 kinase inhibitor AG825 resulted in synergistic growth inhibition, increased G1 arrest, reduced proliferation and increased apoptosis [42]. Interestingly, interaction and crosstalk between IGF-IR and HER2 were noted in BT474 cells, with IGF-IR inhibition reducing HER2 phosphorylation [42, 43]. Cornelissen et al. [15] showed that resistance of HER2-overexpressing breast tumor xenografts to trastuzumab correlated with IGF-IR density, and that this resistance was reversed by addition of IGFBP3 or IGF-IR TKI AG1024. The IGF-IR kinase inhibitor NVP-AEW541 also achieved synergistic inhibition of proliferation when combined with trastuzumab in BT474 cells, with reduced p-Akt and increased p27kip1 expression [44]. NVP-AEW541 reduced proliferation of trastuzumab-resistant BT474 and SKBR3 cells when combined with trastuzumab [43]. The phenolic compound nordihydroguaiaretic acid (NDGA) suppressed IGF-IR and HER2 signaling [45, 46], and induced cell death of trastuzumab-naive and trastuzumab-refractory HER2-over-expressing breast cancer cells [45].

 Several IGF-IR-targeted agents are currently in early phases of clinical development for various types of cancer. IGF-IR mAb ganitumab (AMG 479; Amgen) has elicited responses in treatment-refractory Ewing's sarcoma [47] and is currently being studied in combination with trastuzumab in patients with HER2-positive metastatic breast cancer. Cixutumumab (IMC-A12; ImClone Systems Incorporated) treatment showed a trend toward improved progression-free survival in patients with refractory nonsmall lung cancer who had high baseline IGF-I levels [48]. However, the combination of cixutumumab and EGFR TKI erlotinib that were tested in that study was not tolerable. Cixutumumab is now being tested in combination with lapatinib in patients with metastatic breast cancer. Assessing the dose-limiting toxicity of this combination and careful biomarker analysis will be keys to determining whether dual IGF-IR/HER2 targeting is achievable and what molecular predictors should be measured to rationally select patients to receive this combination. Dalotuzumab (MK-0646, h7C10; Merck) is another IGF-IR mAb being tested in various clinical settings. Previous work showed that dalotuzumab and the EGFR mAb 225 elicited synergistic tumor regression in a xenograft model of the MCF7 breast cancer line [49]. In the context of HER2-positive breast cancer, dalotuzumab is being tested in combination with trastuzumab in xenograft models of trastuzumab resistance. Dual IGF-IR/insulin receptor TKIs BMS-754807 and BMS-754807 (Bristol Myers Squibb) are also being tested in combination with trastuzumab in HER2-positive metastatic breast cancer.

## 7. Conclusions

In summary, IGF-IR expression and signaling are elevated in a subset of trastuzumab-resistant breast cancers. Inhibition of IGF-IR using genetic or pharmacologic approaches shows antitumor activity in cell lines and xenograft models derived from trastuzumab-naïve and trastuzumab-resistant HER2-positive breast cancers. Further *in vivo* analysis of combination IGF-IR and HER2 targeting is required, as only a few studies have shown that pharmacologic inhibition of IGF-IR restores sensitivity to trastuzumab in animal models. In addition, the mechanism of IGF-IR-mediated trastuzumab resistance remains largely unknown. While PI3K signaling appears to play a role, IGF-IR activates multiple downstream pathways that are likely to also be involved in drug resistance. Identification of the signaling molecules that contribute to IGF-I-dependent trastuzumab resistance may provide biomarkers or additional therapeutic targets.

 Several IGF-IR antibodies and kinase inhibitors are now being tested clinically. Based on the preclinical and clinical correlative data presented in this paper, there is rationale for conducting trials that combine IGF-IR inhibitors with trastuzumab in the context of HER2-over-expressing breast cancer that has progressed on prior trastuzumab-containing regimens. Careful biomarker analysis will be an important part of these trials. Therapeutic strategies that co-target IGF-IR and HER2 are likely to achieve benefit in only a subset of trastuzumab-refractory disease, since IGF-independent mechanisms of resistance are also known to occur. In the absence of rational patient selection for trials of IGF-IR-directed agents, a clinical benefit in a subpopulation may be missed. Thus, trastuzumab-refractory breast cancer tissue or patient serum should be examined for possible predictors of response to IGF-IR therapy, such as increased IGF-I levels, reduced IGFBP3 levels, or increased expression or phosphorylation of IGF-IR. Serum markers such as IGF-I or IGFBPs would be easier to obtain, as most patients with metastatic, trastuzumab-refractory disease do not return for biopsy. Validation of these serum markers as true indicators of response will help achieve this important step of rationally selecting which patients should be treated with combination IGF-IR inhibitors plus trastuzumab.

 Strong preclinical data implicating increased IGF-IR signaling as a mechanism of trastuzumab resistance has been collected over the past decade. The next 3–5 years will help to establish whether IGF-IR signaling is a valid clinical predictor of trastuzumab resistance, and/or if IGF-IR is a molecular target whose inhibition can improve response to trastuzumab in patients with HER2-overexpressing metastatic breast cancer.
